# Undifferentiated head and neck tumors: the contribution of immunohistochemical technique to differential diagnosis

**DOI:** 10.1590/S1516-31802003000600005

**Published:** 2003-11-06

**Authors:** Walter Adriano Bianchini, Albina Maria Altemani, Jorge Rizzato Paschoal

**Keywords:** Immunohistochemical, Undifferentiated tumors, Diagnosis, Head neck, Avidin-biotin-peroxidase, Imunoistoquímica, Neoplasias indiferenciadas, Diagnóstico, Cabeça e pescoço, Avidina-biotina peroxidase

## Abstract

**CONTEXT:**

Undifferentiated head and neck and skull base tumors are not unusual. They can arise in mucosa as well as in salivary glands, soft tissues or lymph nodes. Suitable therapy and prognosis for each case depends upon precise histopathological diagnosis.

**OBJECTIVE:**

To evaluate the role of immunohistochemical techniques in determining the conclusive diagnosis. The occurrence of these tumors in our service and the way in which they were distributed according to cell pattern, patient's age and tumor location was also evaluated.

**TYPE OF STUDY:**

Cross-sectional study.

**SETTING:**

Hospital das Clínicas, Universidade Estadual de Campinas, Campinas, São Paulo, Brazil.

**PARTICIPANTS:**

43 biopsies performed between January 1990 and December 1997, diagnosed as undifferentiated head and neck tumors.

**PROCEDURES:**

We applied an immunohistochemical panel in accordance with the avidin-biotin-peroxidase complex method. The final diagnosis was achieved after new analysis in conjunction with biopsies stained using the hematoxylin-eosin technique.

**MAIN MEASUREMENTS:**

This study evaluated undifferentiated tumors in head and neck, and the way in which they were distributed, according to cell pattern, patient's age and tumor location.

**RESULTS:**

The most frequent locations for undifferentiated tumors were the lymph nodes, 20.9%; pharynx and neck, 16.3%; paranasal sinus, 14%; and nose, 11.6%. They were most prevalent during the seventh decade of life (34.9%), and twice as prevalent in men as in women. The immunohistochemical technique allowed conclusive diagnosis for 60.5% of the tumors and was suggestive for 20.9% of the biopsies. The most prevalent cell pattern was round cells (51.2%), followed by epithelioid cells (20.9%), spindle cells (16.3%), myxoid pattern (9.3%) and pleomorphic cells (2.3%).

**CONCLUSION:**

Our results demonstrate the fundamental role of the immunohistochemical technique for conclusive diagnosis of undifferentiated tumors.

## RESUMO

**CONTEXTO:**

As neoplasias indiferenciadas em cabeça e pescoço e base do crânio não são raras. Ocorrem tanto em mucosas como em glândulas salivares, em partes moles e em linfonodos. O diagnóstico histopatológico preciso é fundamental na conduta terapêutica ideal e na caracterização do prognóstico de cada caso.

**OBJETIVOS:**

Avaliar a ocorrência destas neoplasias em nosso serviço, sua distribuição conforme o padrão celular, a idade do paciente e a localização do tumor, avaliando-se a freqüência dos casos em que o exame imunoistoquímico foi decisivo para o diagnóstico diferencial conclusivo.

**TIPO DE ESTUDO:**

Estudo de corte transversal.

**LOCAL:**

Hospital das Clínicas, Universidade Estadual de Campinas, Campinas, São Paulo, Brasil.

**PARTICIPANTES:**

Foram estudadas 43 biópsias de neoplasias indiferenciadas diagnosticadas no ambulatório da Disciplina de Otorrinolaringologia, no período de 1990 a 1997.

**PROCEDIMENTOS:**

Aplicou-se um painel imunoistoquímico conforme o método complexo avidina-biotina-peroxidase (ABC), dependendo da idade dos pacientes, da localização do tumor e do padrão citoarquitetural das células neoplásicas. O laudo final foi emitido após nova análise conjunta com as lâminas coradas pela técnica da hematoxilina e eosina.

**VARIÁVEIS ESTUDADAS:**

Distribuição das neoplasias indiferenciadas de cabeça e pescoço, conforme o padrão celular, a idade do paciente e a localização do tumor.

**RESULTADOS:**

Os locais de ocorrência mais comuns foram os linfonodos, 20.9%; faringe e pescoço, 16.3%; seios paranasais, 14.0% e cavidade nasal, 11.6%. Estas neoplasias foram mais prevalentes na sétima década de vida (34.9%), sendo duas vezes mais prevalentes em homens que em mulheres. O exame imunoistoquímico permitiu o diagnóstico conclusivo em 60.5% dos tumores e o sugeriu em 20.9%. Os padrões citoarquiteturais mais comuns foram: células redondas, 51.2%; células epitelióides, 20.9%; células fusiformes, 16.3%; mixóides, 9,30% e células pleomórficas, 2.3%.

**CONCLUSÃO:**

Esses achados demonstram o papel fundamental do exame imunoistoquímico no diagnóstico conclusivo nestas neoplasias.

## INTRODUCTION

Some tumor groups challenge routine histopathological classification because of the absence of cell morphological differentiation that characterizes their lymphoid, epithelial or mesenchymal origin.^1,2^ These are poorly differentiated or undifferentiated tumors and can occur with relative frequency in the head and neck. They can arise in mucosa as well as in salivary glands, soft tissues or lymph nodes.^3,4^ The diagnosis and classification of such tumors are fundamental because suitable therapy and prognosis for each case depends upon precise histopathological diagnosis.^5,6^

The introduction of the immunohistochemical method, by Coons et al. in 1942,^7^ has become a powerful complementary tool in tumor analysis. It has increased the possibilities for histogenetic diagnosis of undifferentiated tumors. Through the identification of specific cellular components of cell patterns, using a specific panel of monoclonal or polyclonal antibodies, the immunohistochemical method has transformed the diagnosis of these tumors. Diagnoses that used to be made on the basis of subjective information can now be accomplished using objective criteria. However,there are only a few references in the literature to the immunohistochemical technique applied to the identification of undifferentiated head and neck tumors.^8-11^

In this retrospective analysis, we evaluated the occurrence of undifferentiated head and neck tumors in our service and the way in which they were distributed, according to cell pattern, patient's age and tumor localization. The role of immunohistochemical techniques in determining the conclusive diagnosis was also evaluated.

## METHODS

We reviewed 43 biopsies performed in the Departments of Otolaryngology and Head and Neck Surgery of the Universidade Estadual de Campinas, from January 1990 to December 1997, that were diagnosed as undifferentiated tumors. The criteria for inclusion were tumors located in the head and neck, histopathological diagnosis of undifferentiated tumors in sections stained using hematoxylineosin (HE), and sufficient quantity of material in the paraffin section for the immunohistochemical technique to be performed. Tumors with evident differentiation seen in sections stained using HE and specimens with insufficient material for the immunohistochemical technique were excluded.

All the biopsies utilized were fixed in formalin 10%, embedded in paraffin and stained with hematoxylin-eosin. They all had a diagnosis of undifferentiated tumor as seen under optical microscopy, and these tumors were reviewed and distributed according to their cell pattern, into five groups:

1. Round cell tumors
2. Spindle cell tumors
3. Pleomorphic cell tumors
4. Myxoid tumors
5. Epithelioid cell tumors.

We applied an immunohistochemical panel with monoclonal antibodies (Table 1), in accordance with the avidin-biotin-peroxidase complex method (ABC),^12^ and with respect to the patient's age, tumor location and cell pattern. The immunohistochemical panels employed for the analysis of undifferentiated tumors are given in Table 1.

**Table 1:** Immunohistochemical panels employed for the analysis of undifferentiated tumors

**Epithelial**	AE1/AE3	
	MNF116	
	CAM5.2	
	CEA	
	EMA	
**Lymphoid**	LCA	CD 15
	CD 68	CD 30
	CD 45	RO Lysozyme
	CD 20	
**Mesenchymal**	Vimentin	Chromogranin
	Desmin	1^A4^
	Myoglobin	HHF-35
	S 100	HMB-45
	Factor VIII	NSE
	GFAP	

In brief, the biopsy specimens were deparaffinized and rehydrated, and endogenous peroxidase activity was blocked using 3% aqueous hydrogen peroxide in methanol. The slides were sequentially incubated with appropriate non-immune serum and appropriate primary antiserum, overnight at 4° C. This was followed by incubation for 30 minutes with the biotinylated secondary antibody at 27° C and the avidin-biotin complex, before incubation with diaminobenzidine in phosphate buffer.

The final diagnosis was achieved after new microscopic analysis in conjunction with sections stained using the hematoxylin-eosin technique. The results were distributed according to cell pattern, patient's age and tumor location.

## RESULTS

Undifferentiated tumors in the head and neck represented 1.1% of all tumors diagnosed in our service (43 cases out of 3,840 consecutive biopsies). There were 29 male (67.5%) and 14 female cases (32.5%), i.e. a proportion of 2:1, and patients’ ages ranged from 2 to 89 years. These tumors were most prevalent during the seventh decade of life (34.9%) (Table 2). The lymph nodes (20.9%), pharynx and neck (16.3%), paranasal sinus (14%) and nose (11.6%) constituted the majority of sites, followed by oral cavity, ear, skin and larynx (Table 2).

**Table 2:** Immunohistochemical diagnosis and patient's age, cell pattern and location in neoplasias of the head and neck

Variables	CONCLUSIVE	SUGGESTIVE	INCONCLUSIVE	Total
**AGE**				
0-10	3	0	0	3
10-20	2	1	1	4
20-30	4	1	0	5
30-40	1	3	1	5
40-50	1	0	2	3
50-60	5	1	1	7
60-70	9	3	3	15
Over 70	1	0	0	1
**Total**	**26**	**9**	**8**	**43**
**CELL PATTERN** *				
Epithelioid	5	2	2	9
Spindle	1	1	5	7
Myxoid	4	0	0	4
Pleomorphic	0	0	1	1
Round	16	5	1	22
**Total**	**26**	**8**	**9**	**43**
**LOCATION**				
Oral cavity	4	0	0	4
Pharynx	3	4	0	7
Larynx	0	0	1	1
Lymph nodes	8	1	0	9
Nose	2	2	1	5
Ear	0	1	1	2
Skin	1	0	1	2
Neck	3	0	4	7
Paranasal sinus	5	0	1	6
**Total**	**26**	**8**	**9**	**43**

* *p = 0.0357 (chi-squared test).*

The most prevalent cell pattern was round cells (51.2%), followed by epithelioid cells (20.9%), spindle cells (20.9%), myxoid pattern (9.3%) and pleomorphic cells (2.3%) (Table 2).

Diagnostic guidance was possible from 35 (81.4%) biopsies, and conclusive in 26 cases (60.5%). In 8 cases (18.6%), diagnosis was not possible. In the tumors in which diagnosis was possible, we found 12 carcinomas (27.9%), 9 lymphomas (20.9%) and 14 other types of tumors (32.6%) (Table 3).

**Table 3:** Cell pattern and tumor types in neoplasias of the head and neck

Cell pattern /diagnosis	Carcinoma	Lymphoma	Others	Inconclusive	Total
Round	8	8	1	5	22
Epithelioid	4	0	3	2	9
Spindle	0	0	6	1	7
Pleomorphic	0	0	1	0	1
Myxoid	0	1	3	0	4
**Total**	**12**	**9**	**14**	**8**	**43**

## DISCUSSION

The immunohistochemical technique has revolutionized surgical pathology knowledge, because the correct diagnosis of the neoplasia is essential for successful therapy and prognosis. Undifferentiated tumors represent 5-10% of all diagnosed tumors.^13^ In our service, specifically in the head and neck region, the frequency was 1.1%. Undifferentiated tumors were found in all age groups, and were most prevalent in adult and elderly patients, with predominance among the male population (2:1). These data are supported by Azar et al. (1982),^14^ Coindre et al. (1986)15 and Vege et al. (1994).^13^

Round cell tumors, the most prevalent cell pattern found in our sample (51.16%), are a group of highly aggressive malignant tumors composed of relatively small and monotonous undifferentiated cells. These tumors typically arise during childhood but can arise in adults and are often present in undifferentiated tumors. Conclusive diagnosis was most difficult in cases of spindle cell tumors (14.3%), whereas good diagnostic rates were seen in cases of myxoid tumors (100%), round cell tumors (72.7%) and epithelioid cell tumors (62.5%). Conclusive diagnosis was not possible in the single case of pleomorphic cell tumor.

The most frequent location is the lymph nodes,^13^ the majority with the round cell pattern,^7^ where there were five lymphomas, three carcinomas and one inconclusive case. There is greater incidence of lymphoid tumors in this location and more difficulty in achieving conclusive diagnosis by conventional techniques because of the large morphological patterns. Metastases to lymph nodes in the head and neck are also frequent. The immunohistochemical technique was useful in conclusive diagnosis of the tumors in the oral cavity (100%), lymph nodes (88.9%) and paranasal sinuses (83.3%).

Gatter et al.^16,17^ and Coindre et al.^15^ demonstrated greater incidence of lymphomas in undifferentiated tumors in general. In our study, however, carcinomas were the most frequent tumors (27.9%), a result that is similar to findings by Darrouzet et al.^9^ This is probably because carcinomas are the most frequent tumors in the head and neck, which was the subject of our investigation.

Our results showed diagnostic guidance in 35 (81.4%) patients. It was not possible in eight patients (18.6%), probably due to limitations of the technique, antigen changes during tissue fixation or true absence of cellular differentiation.

## CONCLUSIONS

In conclusion, this paper supports the notion that the immunohistochemical technique has a fundamental role in the investigation and definition of undifferentiated tumor origin, thus determining correct guidance for treatment and possibly improving the prognosis for head and neck oncological patients.
